# 恶性胸膜间皮瘤靶向治疗的研究进展

**DOI:** 10.3779/j.issn.1009-3419.2024.102.18

**Published:** 2024-05-20

**Authors:** FU Fen, ZHANG Yang, SHEN Hong

**Affiliations:** 210011 南京，南京医科大学第二附属医院呼吸与危重症医学科; Department of Respiratory and Critical Care Medicine, The Second Affiliated Hospital of Nanjing Medical University,Nanjing 210011, China

**Keywords:** 恶性胸膜间皮瘤, 基因突变, 表观遗传, 信号蛋白, 靶向治疗, Malignant pleural mesothelioma, Gene mutation, Epigenetics, Signaling proteins, Targeted therapy

## Abstract

恶性胸膜间皮瘤（malignant pleural mesothelioma, MPM）是侵袭性极强的罕见胸膜表面恶性肿瘤，危险因素包括吸入石棉、遗传因素、基因突变等。现有的化疗、抗血管生成治疗、免疫治疗的效果均不佳，患者的生存期极短。亟需寻找治疗MPM的潜在靶点，目前发现有基因突变靶点如BRCA1相关蛋白1（BRCA associated protein 1, BAP1）和细胞周期蛋白依赖性激酶抑制剂2A（cyclin-dependent kinase 2A, CDKN2A）等；表观遗传靶点如组蛋白赖氨酸去甲基酶4A[lysine (K)-specific demethylase 4A, KDM4A]和赖氨酸特异性去甲基酶1（lysine-specific demethylase 1, LSD1）等；信号蛋白靶点如葡萄糖调节蛋白78（glucose-regulated protein 78, GRP78）及信号转导和转录激活因子3（signal transducer and activator of transcription 3, STAT3）等。迄今为止，可查询的临床试验有组蛋白甲基转移酶抑制剂Tazemetostat、多聚ADP-核糖聚合酶[poly (ADP-ribose) polymerase, PARP]抑制剂Rucaparib和细胞周期蛋白依赖性激酶4/6（cyclin-dependent kinases 4 and 6, CDK4/6）抑制剂Abemaciclib的II期临床试验，以及靶向间皮素的嵌合抗原受体T细胞免疫疗法（chimeric antigen receptor T-cell immunotherapy, CAR-T）细胞胸腔注射、TEA结构域家族成员（TEA domain family member, TEAD）抑制剂VT3989和IK-930的I期临床试验，显示出一定的临床疗效。

## Abstract (en)

Malignant pleural mesothelioma (MPM) is a rare cancer with high malignancy and aggressiveness on the pleural, caused by the following risk factors including asbestos inhalation, genetic factors, and genetic mutation. The present chemotherapy, antiangiogenic therapy, and immunotherapy methods are ineffective and the survival time of patients is very short. There is an urgent need to find potential therapeutic targets for MPM. At present, it has been found the following types of targets: gene mutation targets such as BRCA associated protein 1 (BAP1) and cyclin-dependent kinase 2A (CDKN2A); epigenetic targets such as lysine (K)-specific demethylase 4A (KDM4A) and lysine-specific demethylase 1 (LSD1), and signal protein targets such as glucose-regulated protein 78 (GRP78) and signal transducer and activator of transcription 3 (STAT3). So far, available clinical trials include phase II clinical trials of histone methyltransferase inhibitor Tazemetostat, poly (ADP-ribose) polymerase (PARP) inhibitor Rucaparib and cyclin-dependent kinases 4 and 6 (CDK4/6) inhibitor Abemaciclib, as well as phase I clinical trials of mesothelin-targeting chimeric antigen receptor T-cell immunotherapy (CAR-T) cell injection in the thoracic cavity and TEA domain family member (TEAD) inhibitor VT3989 and IK-930, and the results of these trials have showed certain clinical efficacy.

恶性胸膜间皮瘤（malignant pleural mesothelioma, MPM）是胸膜腔内间皮细胞产生的一种侵袭性极强的罕见胸膜表面恶性肿瘤。2024年4月国际癌症研究机构报道2022年MPM相关的新发病例30,618例，死亡病例25,372例^[1]^。MPM多数与职业暴露接触石棉有关^[2]^，吸入石棉纤维可引起胸膜炎症、间皮细胞DNA损伤以及巨噬细胞、中性粒细胞的聚集，从而激活致癌因子进而导致肿瘤的发生^[3]^。其他危险因素包括猿猴病毒40感染^[4]^、遗传因素及基因突变如BRCA相关蛋白1（BRCA associated protein 1, BAP1）等^[5,6]^。世界卫生组织（World Health Organization, WHO）第五版肿瘤分类将MPM分为三种组织学亚型：上皮样、肉瘤样和双相型^[7]^。MPM患者早期诊断困难、恶性程度高、疾病进展快，大多数患者在确诊时已处于肿瘤晚期，预后极差，确诊后5年死亡率高达93%^[8]^。

MPM患者确诊后一般接受培美曲塞联合铂类药物一线治疗，疗效极其有限，中位总生存期（median overall survival, mOS）为6-12个月^[9,10]^。MAPS研究^[11]^发现，培美曲塞联合铂类加Bevacizumab可将患者mOS延长至18.8个月。免疫临床试验方面，CheckMate 743^[12,13]^发现使用Nivolumab联合Ipilimumab治疗不可切除MPM患者的mOS为18.1个月，但仅限于非上皮样亚型的MPM患者。DREAM II期试验^[14]^将程序性死亡配体1（programmed death ligand 1, PD-L1）抑制剂Durvalumab联合顺铂和培美曲塞治疗方案进行研究，结果显示在6个月内54例患者中有31例生存且无进展；mOS为18.4个月，12个月OS率为65%，目前III期临床实验正在进行中（NCT04334759）。然而，总体而言，现有的化疗、抗血管生成治疗、免疫治疗的效果均不佳，迫切需要寻找新的治疗方法以延长患者生存期。

近来，已有文献^[15]^报道了MPM免疫治疗的最新研究进展，但是，另外一方面目前临床上尚无MPM靶向治疗药物。当然，MPM的靶点研究也在逐步开展中，MPM的潜在治疗靶点，如靶向基因突变的果蝇zeste基因增强子人类同源物2（enhancer of zeste homolog 2, EZH2）、靶向表观遗传调控（如启动子甲基化异常）、靶向组蛋白修饰物，是MPM治疗的前景。然而大家对它的认知较少，基于已公开的文献和数据，现对MPM的基因突变、表观遗传、信号蛋白、靶向治疗临床试验进展进行综述。

## 1 MPM的基因突变靶向研究进展

据已有文献^[16]^报道，MPM基因突变的主要特点是抑癌基因的失活或缺失，如BAP1、细胞周期蛋白依赖性激酶抑制剂2A（cyclin-dependent kinase 2A, CDKN2A）、神经纤维瘤病2型（neurofibromatosis type 2, NF2）、肿瘤蛋白p53（tumor protein p53, TP53）等。由于抑癌基因突变或缺失使得MPM很难确定合适的干预点用于靶向治疗，而MPM靶向药物的研发成功与寻找该基因信号通路上促癌治疗靶点有一定关系。现针对MPM基因突变靶点的研究报道如下。

BAP1具有抑制肿瘤生长的功能，是MPM的最重要突变靶点，大约60%的MPM患者存在BAP1失活现象^[17,18]^。BAP1是一种泛素羧基末端水解酶，通过减少组蛋白H2A赖氨酸119的单一泛素化降低Polycomb抑制组蛋白甲基转移酶复合物1（Polycomb repressor complex 1, PRC1）的转录抑制功能，PRC1转录抑制功能的降低可减弱PRC1与转录因子或共转录抑制因子的相互作用，进而增强抑癌基因的表达^[19]^。此外，有基础研究^[20]^显示BAP1失活会增加EZH2的表达，而EZH2是组蛋白甲基转移酶PRC2的一个组成部分，是肿瘤驱动因子。事实上，在调控肿瘤方面，PRC2和PRC1之间存在相互作用和协同作用，PRC2的活性可以促进PRC1的招募和功能，而PRC1的泛素化修饰则进一步稳定和维持PRC2的修饰状态，两者共同参与基因调控和维持CDH1等抑癌基因的沉默，导致肿瘤的发生^[21]^。因此，可以推断靶向EZH2是通过抑制PRC1和PRC2的功能并恢复BAP1的肿瘤抑制作用发挥其靶点作用，目前针对EZH2的靶向药物Tazemetostat临床试验正在开展中，详见本文第四部分。

CDKN2A是MPM中又一常见的缺失基因。多区域测序结果显示CDKN2A高频率的纯和缺失会导致肿瘤抑制因子P16INK4A完全缺失^[22]^，而P16INK4A抑制肿瘤的核心是通过与细胞周期蛋白依赖性激酶4和6（cyclin-dependent kinases 4 and 6, CDK4/6）结合并抑制CDK4/6活性进而使细胞周期停滞于G_1_期^[23]^。如何抑制CDK4/6活性从而抑制肿瘤的生长已成为弥补P16INK4A基因缺失的关键。Aliagas等^[24]^通过体内外实验验证了CDK4/6抑制剂增加干扰素信号通路的表达，促进肿瘤抗原递呈过程，进而抑制肿瘤细胞的生长和增殖。不过，也有研究^[25]^报道单药CDK4/6抑制剂在晚期非小细胞肺癌中的整体疗效较差，但与其他药物联合使用可能起到增效的作用。有关CDK4/6抑制剂Abemaciclib用于MPM患者的临床试验正在开展中，详见本文第四部分。

NF2基因缺失大约发生在40%的MPM患者中，它编码神经营养蛋白Merlin。Merlin是一种类肌蛋白ezrin-radixin样肿瘤抑制蛋白，在神经鞘瘤、脑膜瘤和MPM中失活^[26]^。Merlin通过Hippo信号通路和哺乳动物雷帕霉素靶蛋白（mammalian target of rapamycin, mTOR）信号通路调节转录、翻译、泛素化和微小RNAs（microRNAs, miRNAs）生物合成，进而抑制癌症的发生发展^[26]^。最近，关于Hippo信号通路，Papavassiliou等^[27]^报道了它可抑制Yes相关蛋白（Yes-associated protein, YAP）和具有PDZ结合基序的转录共激活子（transcriptional coactivator with PDZ-binding motif, TAZ）过度激活。而YAP和TAZ是转录共激活因子，与TEA结构域家族成员1-4（TEA domain family member 1-4, TEAD1-4）形成YAP/TAZ-TEAD复合体，增强CYR61和CTGF等靶基因的表达，促进癌症发展、转移和耐药^[28]^。而且TEAD同系物在半胱氨酸和巯基上进行自动棕榈酰化靶向发挥促进肿瘤发展的重要作用^[29]^。针对YAP/TAZ-TEAD复合体已成为MPM有前景的治疗策略，临床前研究^[30,31]^证明SWTX、IAG933等可有效抑制YAP/TAZ-TEAD复合体形成，发挥显著的抗肿瘤作用。目前，已开展的针对YAP/TAZ-TEAD靶点的临床试验详见本文第四部分。

miRNAs以细胞周期转录因子为靶标，同时受细胞周期本身的调节，显示出其作为MPM等恶性肿瘤潜在治疗生物标志物的重要性。已有文献^[32]^报道，miR-206为MPM的一个肿瘤抑制因子，通过抑制受体酪氨酸激酶（receptor tyrosine kinase, RTK）-大鼠肉瘤病毒（rat sarcoma, RAS）-丝裂原活化蛋白激酶（mitogen-activated protein kinase, MAPK）-磷脂酰肌醇3-激酶/蛋白激酶B（phosphoinisitide 3-kinase/protein kinase B, PI3K/Akt）-CDK通路中的多种成分从而发挥显著的细胞杀伤作用。当miR-206在MPM高表达时，可阻止细胞周期于G_1_/S期，表现为不可逆转的细胞衰老，从而抑制肿瘤生长。Singh等^[33]^将小鼠异种移植模型随机分为5组，每组分别给予对照组、溶媒对照组、miR-206单药局部用药、Abemaciclib单药口服、miR-206局部用药联合Abemaciclib口服，每周监测小鼠肿瘤负荷。结果显示，与对照组或药物载体相比，miR-206和Abemaciclib单药治疗能显著抑制肿瘤生长，而且联合治疗表现出更为明显的肿瘤抑制作用，证明了胸膜腔内应用miR-206与口服Abemaciclib联合治疗MPM的有效性。

随着研究的深入，越来越多的MPM基因异常被发现，提示与MPM的发生发展相关。肿瘤抑制基因TP53在多种类型癌症中发生突变，它编码的转录因子p53诱导细胞周期停滞和细胞凋亡，抑制肿瘤生长^[34]^。Hiltbrunner等^[35]^研究发现大约17.8%的MPM患者存在TP53的突变。研究^[36]^还显示DNA聚合酶delta 1基因（DNA polymerase delta 1, POLD1）mRNA的高水平表达与MPM患者的OS呈负相关。体外实验^[36]^发现POLD-1缺失可使细胞周期停滞在G_1_期并诱导MPM细胞凋亡。抑制POLD-1表达或许能成为MPM未来的治疗靶点。

## 2 MPM的表观遗传学靶向研究进展

表观基因组由DNA改变（如甲基化）和组蛋白修饰（如乙酰化、甲基化和磷酸化）组成，这些表观遗传修饰均可导致肿瘤抑制基因失活。有研究^[37]^显示，表观遗传失调是MPM的主要诱因之一。介导MPM表观遗传修饰过程与抑癌基因组中的一段二核苷酸区域密切相关，其富含胞嘧啶（cytosine, C）-磷酸（phosphate, P）-鸟嘌呤（guanine, G），简称为CPG岛，长度为300-3000 bp，通常位于基因的启动子区域。CPG岛发生DNA甲基化，可使抑癌基因功能失活^[38]^。DNA甲基化在MPM的发生发展中扮演着十分重要的角色，相关研究报道如下。

泛素样植物同源结构域和无名指结构域1（ubiquitin-like with PHD and ring finger domains 1, UHRF1）是一种多结构域蛋白，主要作用是将DNA甲基转移酶招募到新合成的DNA上^[39]^。它在多种癌症中高表达，可致DNA甲基化和组蛋白修饰^[40,41]^。Reardon等^[42]^研究结果显示，MPM细胞中的UHRF1 mRNA和蛋白质水平显著高于正常间皮细胞，其表达水平与患者的OS呈负相关，UHRF1高表达患者的mOS仅为8.7个月，UHRF1低表达患者的mOS可达22.2个月。通过构建小鼠MPM模型发现特异性蛋白1（specificity protein 1, SP1）抑制剂普卡霉素或人双微基因2（human double minute 2, HDM2）抑制剂可以通过增强p53信号传导从而抑制MPM小鼠中UHRF1过表达，从而抑制MPM的生长。因此，靶向UHRF1或许能显著提高患者的生存期，但UHRF1靶向药物的临床试验尚未开展，需进一步的探索。

组蛋白赖氨酸去甲基酶4A[lysine (K)-specific demethylase 4A, KDM4A]能特异性催化组蛋白H3赖氨酸K36去甲基化，通过参与细胞生长等方式对细胞有丝分裂产生间接影响，具有调控细胞凋亡、DNA修复、剪接、自我更新等作用，在癌症的发展过程中具有重要作用^[43]^。KDM4A在MPM中过度表达，KDM4A抑制剂通过抑制细胞周期进程和诱导细胞凋亡从而抑制肿瘤细胞生长。通过构建KDM4A敲除小鼠模型，Lapidot等^[44]^发现KDM4A缺失可抑制MPM细胞生长并降低癌细胞的活力。目前靶向KDM4A尚处于基础研究阶段，能否研究出靶向药物仍未知，需要进一步的研究和探索。

赖氨酸特异性去甲基化酶1（lysine-specific demethylase 1, LSD1）是一种依赖于黄素腺嘌呤二核苷酸的组蛋白去甲基化酶，可去除组蛋白H3赖氨酸4和9的单甲基化和双甲基化，抑制靶基因的转录，从而发挥致癌作用^[45]^。MPM的预后与上皮间充质转化（epithelial-mesenchymal transition, EMT）有关，而EMT可能是导致化疗耐药的原因之一，提示靶向EMT或许可有效治疗MPM患者。Wirawan等^[46]^研究发现LSD1正是通过黏着斑激酶（focal adhesion kinase, FAK）-Akt-糖原合酶激酶3β（glycogen synthase kinase 3β, GSK3β）通路激活EMT，抑制LSD1可调节乳脂球蛋白E8（milk fat globulin protein E8, MFGE8）和Snail表达的正反馈环来抑制EMT，并使MPM细胞对顺铂诱导的细胞凋亡敏感，因此有学者提出LSD1抑制剂联合顺铂可作为治疗MPM的新方法。LSD1抑制剂反苯环丙胺（tranylcypromine, TCP）已在血液系统恶性肿瘤中开展临床试验（NCT02717884、NCT02273102），结果显示TCP可提高全反式维甲酸治疗急性髓系白血病患者的疗效，而针对MPM的临床试验尚未开展。

## 3 MPM的信号通路蛋白靶向研究进展

信号通路中上游蛋白对下游蛋白活性的调节（包括激活或抑制作用）主要是通过添加或去除磷酸基团，从而改变下游蛋白的立体构象，在恶性肿瘤发生发展中发挥重要作用。现已发现多种信号通路蛋白可致MPM的生长和侵袭，现将相关研究报道如下。

此前有研究^[47,48]^报道葡萄糖调节蛋白78（glucose-regulated protein 78, GRP78）在MPM和其他多种癌症中升高，可激活肿瘤细胞生长和增殖，对常规使用的化疗药物（如顺铂、紫杉醇、阿霉素）耐药，靶向GRP78的研究正在进行中。BOLD-100是一种静脉内给药的无菌溶液，含有钌基小分子，其在癌细胞中的作用机制复杂，其主要途径之一是通过caspase-8激活阻断GRP78上调，激活下游未折叠蛋白反应（unfolded protein response, UPR）的信号通路，进而导致细胞凋亡^[49]^。体外实验^[50]^结果显示，BOLD-100显著抑制MPM细胞系的生长。2020年在美国、加拿大、韩国13家医院联合开展BOLD-100与FOLFOX联合治疗的IIa期临床试验（NCT04421820），2023年6月，美国临床肿瘤学会（American Society of Clinical Oncology, ASCO）年会上公布了晚期胃癌和胆道癌II期研究的初步数据，相比于标准治疗患者，BOLD-100联合治疗组中位无进展生存期（median progression-free survival, mPFS）和mOS均明显延长^[51]^。因而，美国食品药品监督管理局（Food and Drug Administration, FDA）于2023年批准BOLD-100用于治疗胃癌和胰腺癌患者。我们期待有针对MPM的临床试验开展以提高治疗MPM的疗效。

Fibulin-3是一种细胞外基质（extracellular matrix, ECM）蛋白，参与组织ECM支架并促进正常结缔组织中的细胞黏附^[52]^。研究^[53]^发现，Fibulin-3在MPM患者的胸腔积液中升高，其升高与肿瘤的生长与侵袭密不可分。Roshini等^[54]^通过构建小鼠MPM模型首次证明了Fibulin-3通过激活PI3K/Akt信号通路从而直接促进MPM的侵袭行为和肿瘤生长。虽然目前Fibulin-3在MPM中的作用机制尚未完全阐明，但总体研究结果显示Fibulin-3可能是肿瘤靶向治疗的潜在研究方向。

信号转导和转录激活因子3（signal transducer and activator of transcription 3, STAT3）是常见的信号转导蛋白。在生理条件下，它会响应各种细胞因子和生长因子而短暂激活，调节控制细胞增殖、存活和自我更新的基因表达^[55,56]^。Lapidot等^[57]^通过基因表达谱及构建小鼠MPM模型发现STAT3通路激活可诱导MPM肿瘤微环境中的免疫抑制细胞的产生，研究还发现STAT3抑制剂阿托伐醌和乙胺嘧啶等药物可通过靶向STAT3蛋白抑制MPM肿瘤细胞生长，并可以减少MPM微环境中的免疫抑制细胞，如调节性T细胞等。该报道首次将STAT3蛋白认为是MPM模型中具有独特免疫效应的细胞生长调节剂，并提出了针对该靶点靶向治疗的研发的可行性。

骨桥蛋白（osteopontin, OPN）是由巨噬细胞、间质细胞和上皮细胞产生的一种高度磷酸化的基质细胞蛋白，可作为MPM的诊断生物标志物^[58,59]^。骨桥蛋白有多种机制促进肿瘤细胞的增殖，包括与整合素或CD44结合，进而刺激FAK-Src-Rho通路，促进癌细胞的黏附和存活；PI3K/Akt通路的激活促进肿瘤血管生成及肿瘤细胞募集等。因此，抑制OPN信号转导可能是抑制间皮瘤细胞生长的潜在靶点^[60,61]^。目前对于OPN在治疗MPM患者方面仍处于基础实验阶段，需要进一步的研究和探索。

间皮素（mesothelin, MSLN）是膜结合的细胞外蛋白，是一种能与黏蛋白16结合的癌症相关抗原^[62]^。MSLN在间皮瘤、胰腺癌和卵巢癌等恶性肿瘤中过度表达^[63]^。在卵巢癌耐药机制的研究^[64]^中发现MSLN可通过激活PI3K/Akt和MAPK信号通路使肿瘤细胞逃逸紫杉醇诱导的细胞凋亡。还有研究^[62]^表明MSLN的表达与白介素-6（interleukin-6, IL-6）密切相关，MSLN过表达能够激活IL-6介导的信号转导，从而促进肿瘤细胞的增殖。而且MSLN高表达与患者的生存时间呈负相关^[65]^。因此，以上研究均提示MSLN是MPM靶向治疗的重要靶点，有关该方面的临床研究进展详见本文第四部分。

## 4 MPM的临床试验研究进展

如前所述，尽管有不少针对MPM的治疗靶点研究，但绝大数仍处于基础实验和动物研究阶段，临床试验仍处于起步阶段。目前，从全球性临床试验登记平台（http://ClinicalTrials.gov）可查找到的临床试验如下文。

Tazemetostat是2020年于美国上市的一种选择性的组蛋白甲基转移酶抑制剂即EZH2抑制剂。2013至2016年间法国有研究中心开展Tazemetostat用于治疗复发性或难治性B细胞非霍奇金淋巴瘤或晚期实体肿瘤患者的I期临床试验，结果显示安全性及抗肿瘤活性良好。2020年Tazemetostat被FDA批准用于滤泡性淋巴瘤患者的治疗。随后，2019年法国、英国和美国的16家医院联合开展的一项单臂、多中心、开放标签的II期临床研究^[66]^，纳入了复发性或难治性BAP1失活的74例MPM患者。第一部分研究纳入13例患者，给药方案为口服800 mg d1 qd，第2天开始为800 mg bid，结果显示Tazemetostat及其代谢物在用药第15天时药物达峰浓度平均值为829 ng/mL，食物对其吸收率、延长吸收率以及药物暴露特性没有影响，显示出良好的安全性和耐受性。在第二部分研究中，采用两阶段Green-Dahlberg设计，纳入BAP1失活的61例MPM患者，从第1天开始，Tazemetostat即口服800 mg bid，如患者第一次出现3级治疗相关不良事件剂量降为口服600 mg bid，第二次出现剂量降为400 mg bid，第三次还出现严重不良反应则研究终止。中位随访了35.9周后，研究结果显示第12周时MPM患者的疾病控制率（disease control rate, DCR）为50%，mPFS为18周，mOS为36周。总体来说，Tazemetostat虽可一定程度延长MPM患者的mOS和mPFS，但整体疗效仍不佳，仍需继续探索更好的治疗策略。

Rucaparib可通过抑制多聚ADP-核糖聚合酶[poly (ADP-ribose) polymerase, PARP]合成来阻断修复受损DNA的酶，进而阻止携带BAP1缺失的癌细胞进行DNA修复，促使癌细胞死亡并减缓或抑制肿瘤的生长。2014年FDA批准其用于卵巢癌和乳腺癌的靶向治疗。2018年英国肺脏基金会资助英国莱斯特大学开展了针对复发MPM患者的多臂分层MiST临床试验，其基于患者生物标志物测试结果制定相应的治疗方案。随后，2021年英国莱斯特癌症研究中心发起的一项单臂、开放标签的IIa期MiST1临床试验^[67]^，纳入了26例MPM患者，23例患者BAP1阴性。患者口服Rucaparib 600 mg bid治疗，4周为1个治疗疗程，持续24周。24周后的结果显示12周时的DCR为58%，24周时的DCR为23%，随访结果显示患者的mPFS为17.9周，OS为41.4周。Rucaparib治疗对于MPM患者的mPFS和OS改善不明显，仍需继续探索。

Abemaciclib是2017年于美国上市的一种口服可利用的生物小分子CDK4/6抑制剂^[68]^。2022年英国癌症研究中心发起了一项单臂、开放标签、II期MiST2临床试验^[69]^，该试验纳入P16INK4A阴性的成人MPM患者，旨在评估复发性MPM的靶向治疗。纳入的P16INK4A阴性的26例MPM患者口服Abemaciclib 200 mg bid治疗，持续24周。24周后的结果显示患者12周时的DCR为54%，80%的患者肿瘤体积有不同程度缩小。通过随访发现，mPFS为128 d，mOS为217 d。MiST1和MiST2试验结果显示患者12周时的DCR均约50%，疗效均有限，目前MiST3试验尚未开展，期待MiST3试验能够有新的突破。

VT3989是一种TEAD棕榈酰化抑制剂，基础试验结果已证明其可高效阻断YAP，抑制肿瘤细胞生长。2023年ICMJE公司开展的一项开放标签、剂量递增的I期临床试验纳入了67例化疗后进展的实体肿瘤患者^[70]^，29例为MPM患者（NCT04665206）。患者分别进行VT3989 25、50、100、150、200 mg qd连续口服和间断口服，3-4周为1个治疗疗程。结果显示安全性和耐受性良好，且没有剂量毒性，但只有7例患者部分缓解，结果不尽如人意。该药物2期推荐剂量临床试验正在进行中^[70]^。另一种TEAD棕榈酰化抑制剂IK-930，通过靶向Hippo通路抑制肿瘤生长^[71]^。目前，关于IK-930的安全性的I期临床试验正在招募晚期实体肿瘤及MPM患者（NCT05228015）。近年来，靶向YAP/TAZ-TEAD信号轴是肿瘤治疗的新策略，TEAD抑制剂已显示出抗肿瘤潜力，但大多处于临床I期试验阶段，期待TEAD靶向药新的疗效数据出现。

嵌合抗原受体T细胞免疫疗法（chimeric antigen receptor T-cell immunotherapy, CAR-T）是一种治疗肿瘤的新型精准靶向疗法。2021年美国癌症研究中心首次开展了一项开放标签、单中心I期试验^[72]^，纳入了25例先前接受过化学治疗的复发性MPM患者，在胸膜腔内注射靶向MSLN的CAR-T细胞，剂量为0.3-60 mol CAR-T细胞/kg。靶向MSLN的CAR-T细胞的制造过程如下：从患者外周血或骨髓中提取T细胞，构建包含MSLN、scFv和CD28/CD3的转基因载体，最后通过逆转录病毒载体将构建好的转基因载体导入T细胞。在第一剂CAR-T细胞治疗后，疗效显著且未发生严重不良事件的患者考虑输注第二剂CAR-T细胞，最多注射2次CAR-T细胞。治疗过程中6例患者出现3级不良反应。最终结果显示患者输注CAR-T细胞后的mOS为17.7个月，12个月OS率为74%。其中18例MPM患者在接受CAR-T细胞局部治疗后，再接受Pembrolizumab静脉治疗的患者mOS可达23.9个月，因此该研究也证明了阻断程序性死亡受体1（programmed cell death 1, PD-1）可协同增强CAR-T细胞的抗肿瘤作用，显示出MSLN CAR-T细胞胸腔局部注射联合静脉使用PD-1抑制剂或许可有效治疗MPM患者。

## 5 展望与小结

由于目前缺乏有效的生物学标志物筛查有石棉接触史或其他诱因的高危患者，大多数MPM患者确诊时已为晚期。目前，MPM虽有化学治疗、抗血管生成治疗、免疫治疗等治疗方法，可一定程度延长患者的PFS和OS，但仍缺乏有效延长生存期的治疗药物。目前，对于MPM的治疗靶点仍处于基础研究或临床前研究阶段，所开展的临床试验较少，多为I和II期，取得的一定程度有意义的临床结果，如MSLN CAR-T细胞胸腔局部注射联合静脉使用PD-1抑制剂可显著延长MPM患者的生存期，但大多数临床试验疗效尚有限，仍需继续探索和研究MPM的肿瘤发生机制以及基因组改变和炎症的作用，增加对MPM生物学机制的研究，并展开更多临床试验，以期找到更有效的治疗方法以提高患者的生存时间及生存质量。
